# Single nucleotide markers of D-loop for identification of Indian wild pig (*Sus scrofa cristatus*)

**DOI:** 10.14202/vetworld.2015.532-536

**Published:** 2015-04-24

**Authors:** Gaurav Kumar Srivastava, Nidhi Rajput, Kajal Kumar Jadav, Avadh Bihari Shrivastav, Himanshu R. Joshi

**Affiliations:** Centre for Wildlife Forensic and Health, Nanaji Deshmukh Veterinary Science University, Jabalpur, Madhya Pradesh, India

**Keywords:** D-loop, Indian wild pig, single nucleotide markers, species identification

## Abstract

**Aim:**

Partial fragment of D-loop region extending from 35 to 770 were compared with corresponding sequences of 16 wild pigs and 9 domestic pig breeds from different parts of the world for detection of single nucleotide polymorphism (SNP) markers in the region. The paper also reappraises SNP markers from two fragments of cytochrome b gene and a fragment 12S rRNA gene distinguishing the Indian wild pig from other pig species of the world.

**Materials and Methods:**

Deoxyribonucleic acid (DNA) was isolated from 14 and 12 tissue samples of Indian wild and domestic pigs, respectively, collected from Central India for characterization of the D-loop DNA sequences using universal primers. The sequences obtained were aligned along with the retrieved sequences to analyze species-specific SNP marker.

**Results:**

A total of 58 mitochondrial D-loop gene sequences of pig races were aligned to identify 1349 polymorphic sites in the fragment from nucleotide positions 35-770 bp and four SNP markers were identified to differentiate Indian wild pig from all the sequences investigated in this study. With the inclusion of cytochrome b gene and 12S rRNA gene fragments, the present study contributes to the total 15 SNP markers, which have been identified in the mitochondrial fragment of 1936 bp for identification of Indian wild pig.

**Conclusion:**

SNP markers have advantages over other marker types and do not require subsequent standardization to compare data across studies or laboratories.

## Introduction

Wild pig (*Sus scrofa*) distributed across many countries of Europe, Asia, and Africa belongs to the Suidae family. It is a common game animal and exploited for substantial consumption. It is a protected species in many countries including India. The Indian wild pig is a separate sub-species (*Sus scrofa cristatus*) from the domestic pig and is under Schedule-III of the Indian Wildlife (Protection) Act, 1972. On the other hand, domestic pig *(Sus scrofa domesticus*) is an important farm animal all over the world and is continuously harvested since its domestication for more than 8000 years back [1]. In India, pig farming is a substantial source of income for marginal farmers as pigs are usually reared in the open system, which involves zero cost of feeding and housing. The growing need results into extensive poaching of wild pig for its meat and in the absence of substantial genetic database, the protocols for differentiation of Indian wild and domestic pig needs to be improved to further aid the law enforcing agencies for prosecution of the poachers in the Hon’ble Court of law.

DNA sequence variation in mitochondrial DNA has been established as an asset for identification of confiscated biological samples of Swine origin [2,3]. Briefly, the procedure involves selective amplification of a gene using the universal primer and sequencing in an automatic DNA analyzer. The raw sequences are matched with the array sequences available in the public domain. However, it must be noted that the assigning a sequence deduced from a seized sample using homologous sequences (based on percentage similarity) from NCBI may lead to erroneous results; so generating sequences from at least three genes are recommended to eliminate chances of species misidentification [4]. Moreover, representative sequences originating from Indian wildlife species in the GenBank are very few. Hence, generating known sequences of Indian wildlife species origin are important for submission in GenBank.

In this study, the partial fragment of D-loop region extending from 35 to 770 was compared with corresponding sequences of 16 wild pigs and 9 domestic pig breeds from different parts of the world for detection of single nucleotide polymorphism (SNP) markers in the region. The paper also reappraises SNP markers from two fragments of cytochrome b gene and a fragment 12S rRNA gene distinguishing the Indian wild pig from other pig species of the world.

## Materials and Methods

### Ethical approval

Necessary permissions from the University Ethical Committee were taken to conduct the research work at Centre for Wildlife Forensic and Health, Jabalpur.

### DNA isolation and DNA sequencing

We used 14 and 12 tissue samples of Indian wild and domestic pigs, respectively, collected from Central India for characterization of the D-loop DNA sequences. Meat samples were collected from domestic pigs from local slaughterhouses and from Indian wild pigs during post mortem examination. DNA was extracted using DNeasy Blood and Tissue Kit (QIAGEN, Germany) in a final elution volume of 200 µl as per manufacturer’s instructions. These samples were screened and confirmed by polymerase chain reaction-restriction fragment length polymorphism (PCR-RFLP) assay, which has been reported in our recent study for differentiation of Indian wild pig from exotic and local domestic pigs [2]. DNA was subjected to routine PCR amplification by using universal primers for D-loop region (Forward- 5’-AGG AGACTAACTCCGCCAT-3’, Reverse-5’- CGCGGATACTTGCATCTGT3’) [5] in a gradient thermal cycler (Eppendorf, India) in a final volume of 50 µl containing 40-50 ng of extracted DNA, 10 mM Tris-HCl (pH 8.3), 50 mM KCl, 1.5 mM MgCl_2_, 0.2 mM of each dNTP, 1 unit of *Taq* polymerase (Applied Biosystems), 0.5 µg/µl bovine serum albumin, and 20 pmol of each primer. The amplification parameters were 94°C for 15 min followed by 35 cycles of 94°C for 30 s, 60°C for 45 s, and 72°C for 1 min with final extension at 72°C for 10 min prior to a 4°C hold. The PCR products were resolved on 1% agarose gel and visualized under UV light in the presence of ethidium bromide dye.

Unused primers and dNTPs in the PCR products were cleaned by treating the PCR products with Exonuclease I *(ExoI*) and srimp alkaline phosphatase (SAP) as per the instructions of supplier (ExoSAP-IT, USB, Cleveland, Ohio). The cleaned PCR products were sequenced (Applied Biosystems Genetic Analyzer) using 3.1 sequencing kit (Applied Biosystems) from both ends.

### Sequence analysis

The sequences obtained from the Indian wild and domestic pig samples were aligned using Clustal W [6] in Molecular evolutionary genetics analysis [6,7] along with the retrieved sequences of 17 exotic wild pigs, 8 exotic domestic pig breeds and 5 sequences of wild pig of South East Asia, and 2 sequences of domestic pig from the Indian sub continental region from GenBank (Figure-1). Aligned sequences were analyzed for species specific SNP marker.

**Figure-1:**
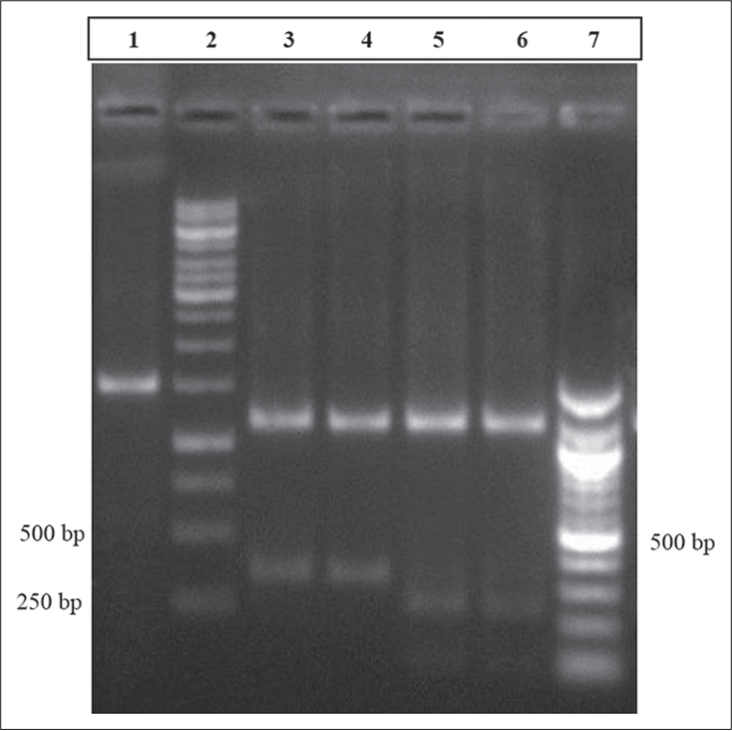
Gel electrophoresis of mitochondrial D-loop gene fragments of Indian wild and domestic pig sequences digested with BsrI Lane 1: Undigested product Lane 2: 1 Kb DNA ladder Lane 3,4: Digested PCR product of domestic pig Lane 5,6: Digested PCR product of Indian wild pig Lane 7: 100 bp DNA ladder.

**Figure-1:**
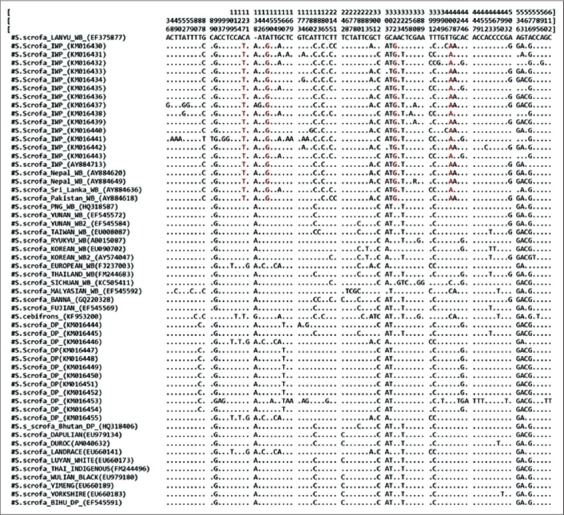
Sequence variation in partial fragment (735 bp) of D-loop region. Three digit numeric values at the top is the position of variable nucleotide in the fragment. Dots (“.”) are for indicating “similarity” for all the identical nucleotides. Alphanumeric in the brackets are the NCBI GenBank accession numbers of DNA sequence. Single nucleotide polymorphism markers are highlighted and bracketed by red colored dots.

### RFLP

**Figure-2:**
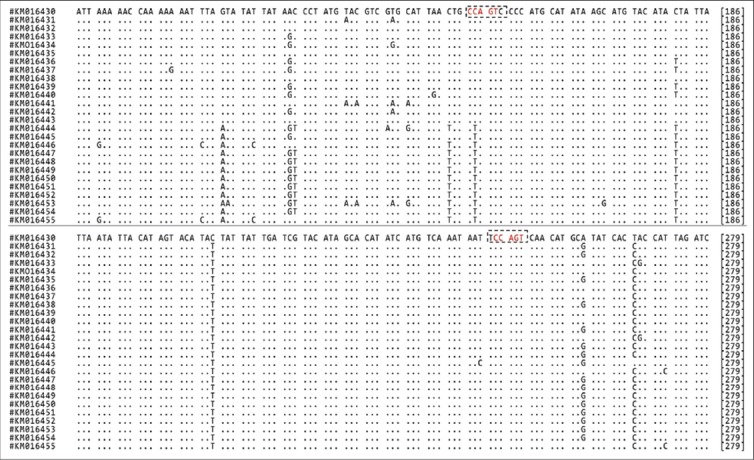
Alignment of mitochondrial D-loop gene fragments of Indian wild and domestic pig. The restriction sites of endonuclease BsrI (5’CCAGT3’) are highlighted and bracketed. “.” for indicating “similarity” for all identical nucleotide.

The newly obtained sequences of Indian wild pig and domestic pig were aligned to analyze intra-species and species-specific variations (Figure-2). *Bsr1 (*5’CCAGT3’) (Fermantas, Shanghai, China) was selected for RFLP analysis based on species-specific sequence variations. PCR products were digested at 37°C for 6 h in a total volume of 20 µl which contained 3 µl of template, 5U of restriction enzymes, and 2 µl digestion buffer. The digested products were visualized by 3% Agarose gel electrophoresis and stained with ethidium bromide.

## Results and Discussion

All samples were successfully amplified to expected size of ~1500 bp. The sequences generated from the amplified PCR products of Indian wild pigs matched with 99% homology among them and had 96-97% homology with the corresponding sequences of the domestic pig. All the obtained sequences of Indian wild pig were submitted to GenBank under accession number from KM016430 to KM016443 while sequences of the domestic pig were submitted under accession no. KM016444 to KM016455. There were two restriction sites for *Bsr1* in the mitochondrial D-loop gene fragment of Indian wild pigs while there was only one restriction site for the enzyme in domestic pig sequences (Plate-2). The *Bsr1* restriction enzyme generated three fragments for Indian wild pig (97, 278 and 1125 bp) and two fragments in the domestic pig (375 and 112 bp) (Figure-1). The four species-specific SNP markers were identified within the investigated sequences (Plate-1). The nucleotide positions were generated from complete mitochondrial sequence of Lanyu wild boar (GenBank Accession no. EF375877). The relative frequency of mispairing was higher in pyrimidine-pyrimidine than purine-purine and transversions in the mitochondrial fragments of Indian wild pig.

SNP detection technologies are used to scan for new polymorphisms and to determine the allele(s) of a known polymorphism in target sequences. DNA sequencing as a scanning method for SNPs is highly competitive when compared to other methods such as denaturing gradient gel electrophoresis, chemical cleavage of mismatches, single-strand conformation polymorphism, cleavage fragment length polymorphism analysis, *minimal residual disease*, T4 endonuclease VII cleavage of heteroduplex DNA, denaturing high-performance liquid chromatography, sequencing by hybridization [8].

The mitochondrial genome is considered to be mutation hotspot [9]. The combination of mtDNA instability and a high fixation rate means that the mutation rate in mitochondrial DNA is very high. Mutations have been reported to be fixed in the mitochondrial genomes of animal cells at a rate which is about 10 times greater than occurring in equivalent sequences in the nuclear genome [10]. Three transitions and one version were identified in the mitochondrial fragment of Indian wild pig within the investigated region of 35-770 bp. With the inclusion of present study, a total of 15 SNP markers have been identified in the mitochondrial fragment of 1936 bp for identification of Indian wild pig, which includes cytochrome b (421 and 398 bp) and 12S rRNA (382 bp) genes, as observed in previous studies [3,11,12] (Table-1). Among the SNP markers, 60% of mutations were pyrimidine to pyrimidine and 27% were purine to purine and only 13% were purine to pyrimidine or vice versa. Lower frequency of transversions in the mitochondrial fragments can be explained by endosymbiotic theory (i.e. common ancestral origin of mitochondria and bacteria) [13] and in the bacterial population, transversions arise through purine-purine mismatches [14].

**Table-1:** List of SNP markers in the four different fragments of the mitochondrial genome of Indian wild pig as observed in the previous and present studies.

Gene	Position of nucleotide	SNP	Accession numbers included in the study	References
Cytochrome b (421)	15689 15693 15725 15785 15825 15927 15949	T>C A>G G>A A>C C>T T>C T>C	JN039027, JN039028, JN039029, JN039030, JN039031, JX411960, DQ315600, EF375877, AB015075, FJ237003, AF304201, AY574047, AB015083, GU135837, GU135826, GU135824, GU135821, GU135820, GU135808, GU135805, GU135794, Z50087, AY237484	[12]
Cytochrome b (398)	15452 15512 15572	C>T C>T T>C	EF375877, KJ652498, KJ652498, KJ652500, J652501, KJ652502, JN242241, FJ190159, FJ190161, ZS0087, AF304201, FJ237003, GU135794, GU135808, GU135820, AY237484, GU135826, GU135805, DQ315600, GU135837, AB015083, AB015075, EU000390, DQ315603, GQ338962, GQ338963, GQ338964, GQ220328, EF 545569, EF545592, KC505411, KF952600, AY830167, AY920905, AF486874, KC505410, JN601074, AY574046, AF486858, JN406914, KC469587, JN601069, JN601072	[3]
12S rRNA (382)	1870	T>C	EF375877, KP205285, KP205301-KP205321, KC505411, EF545579, EF545572, EF545584, EF545585, GU147934, EF545571, FJ237003, AF304201, EF545592, KF952600, KF926379, EU090702, KM203891, EU117375, AF304203, EU333163, KP205286- KP205300, EF545591, KJ651276, GQ220328, KM073256, KC505410, AF486874, JN601075, AY574046	[11]
D-loop (735)	149 322 407 675	A>G A>G T>A C>T	KM016430 to KM016443, KM 016444 to KM016455, EF375877, AY884713, AY884620, AY884649, HQ318587, AY884636, AY884618, EF545572, EF545584, EU008087, AB015087, EU090702, AY574047, FJ237003, FM244683, KC505411, EF545592, GQ220328, EF545569, JQ668033, KF953200, HQ318406, EU979134, AM040632, EU660141, EU660173, FM244496, EU979180, EU660189, EU660183, EF545591, DQ779454	Present study

SNP=Single nucleotide polymorphism

## Conclusion

*Bsr1* restriction enzyme-based PCR-RFLP assay has been designed for identification of Indian wild pig involving SNP markers. More genotyping tests such as species-specific primers, SNaPshots, etc., can be designed with the identification of SNP markers. SNP markers have several advantages over other marker types, chiefly the SNPs’ reproducibility within and across hardware platforms, capacity for high throughput genotyping [15], and sequence-based data that do not require subsequent standardization to compare data across studies or laboratories.

## Authors’ Contributions

KKJ and ABS designed the study. GKS and NR executed the study. KKJ and HRJ analyzed the sequence data. KKJ, NR, and ABS drafted and revised the manuscript for critical scientific corrections. All authors read and approved the final manuscript.

## References

[1] Larson G, Dobney K, Albarella U, Fang M, Matisoo-Smith E, Robins J, Lowden S, Finlayson H, Brand T, Willerslev E, Rowley-Conwy P, Andersson L, Cooper A (2005). Worldwide phylogeography of wild boar reveals multiple centers of pig domestication. Science.

[2] Jadav K.K, Shrivastav A.B, Rajput N (2013). Development of molecular tools to differentiate Indian wild pig (*Sus scrofa cristatus*) meat from exotic and local domestic pig meat. Vet. World.

[3] Jadav K.K, Shrivastav A.B, Rajput N, Joshi H.R (2014). Cytochrome b gene based phylogeny and genetic differentiation of Indian wild pig (*Sus scrofa cristatus*). Indian Res. J. Genet. Biotechnol.

[4] Thakur M (2014). Role of DNA forensics in curbing illegal wildlife trade. WWF newsletter (Panda). Illegal Wildlife Trade in India (Special Issue).

[5] Yu G, Xiang H, Wang J, Zhao X (2013). The phylogenetic status of typical Chinese native pigs: Analysed by Asian and European pig mitochondrial genome sequences. J. Anim. Sci. Biotechnol.

[6] Thompson J.D, Higgins D.G, Gibson T.J, Clustal W (1994). Improving the sensitivity of progressive multiple sequence alignment through sequence weighting, position specific gap penalties and weight matrix choice. Nucleic Acids Res.

[7] Tamura K, Stecher G, Peterson D, Filipski A, Kumar S (2013). MEGA6: Molecular evolutionary genetics analysis version 6.0. Mol. Biol. Evol.

[8] Pui-Yan K, Chen X (2003). Detection of single nucleotide polymorphisms. Curr. Issues Mol. Biol.

[9] Strachan T, Andrew P.R (1999). Human Molecular Genetics.

[10] Brown W.M, George M, Wilson A.C (1979). Rapid evolution of animal mitochondrial-DNA. Proc. Natl. Acad. Sci. U.S.A.

[11] Jadav K.K, Shrivastav A.B, Rajput N, Mandal S, Shrivastava G (2014). Application of 12S rRNA gene sequences for identification of Indian wild pig (*Sus scrofa cristatus*). J. Meat Sci. Technol.

[12] Gupta S.K, Kumar A, Hussain S.A, Vipin, Singh L (2012). Cytochrome b based genetic differentiation of Indian wild pig (*Sus scrofa cristatus*) and domestic pig (*Sus scrofa domestica*) and its use in wildlife forensics. Sci. Justice.

[13] Martin W, Mayo R, Thorsten K, Thorsten T, Christian W, Sven G, Tal D (2012). Modern endosymbiotic theory: Getting lateral gene transfer in-to the equation. J. Endocytobiosis Cell Res.

[14] Fersht A.R, Knill-Jones J.W (1981). DNA polymerase accuracy and spontaneous mutation rates: Frequencies of purine. purine, purine.pyrimidine, and pyrimidine.pyrimidine mismatches during DNA replication. Proc. Natl. Acad. Sci. U.S.A.

[15] Melton L (2003). Pharmacogenetics and genotyping: On the trail of SNPs. Nature.

